# Seasonal Microbial Community Characteristic and Its Driving Factors in a Copper Tailings Dam in the Chinese Loess Plateau

**DOI:** 10.3389/fmicb.2020.01574

**Published:** 2020-07-10

**Authors:** Tong Jia, Tingyan Guo, Yushan Yao, Ruihong Wang, Baofeng Chai

**Affiliations:** Shanxi Key Laboratory of Ecological Restoration on Loess Plateau, Institute of Loess Plateau, Shanxi University, Taiyuan, China

**Keywords:** soil bacterial, soil fungal, structure and function, seasonal dynamics, copper tailings dam

## Abstract

A combined soil bacterial and fungal community survey was conducted for a copper tailings dam in the Chinese Loess Plateau. We investigated the seasonal differences in the composition and function of soil microbial community to examine the key environmental factors influencing soil microorganisms during restorative ecological processes. Significant seasonal differences were found in the community structure of both bacterial and fungal communities. Bacterial community abundance and fungal community (Shannon index) measurements were highest in summer. Soil nitrite nitrogen (NO_2_^−^-N) was the dominant factor influencing both bacterial and fungal communities. The bacterial community composition was significantly affected by NO_2_^−^-N and ammonium nitrogen (NH_4_^+^-N) in spring, and fungal community structure was significantly affected by soil water content in autumn. Moreover, the fungal community exhibited significant functional feature differences among seasons, whereas bacterial community functional groups remained similar. This study aimed to clarify the adaptation response of microbes applying different approaches used in ecological restoration approaches specific to mining areas, and to identify the natural biofertility capacity of the microbial communities that colonize soil ecosystems.

## Introduction

Microorganisms are important in the exchange of mass and energy among the atmosphere, lithosphere, hydrosphere, and biosphere, and they play important roles in global ecological restoration, environmental variation and monitoring, pollution treatments, and biological conservation (Zhu et al., 2017). Microorganisms are ubiquitous throughout all environments, and they are especially important in the restoration of degraded ecosystems in mining areas (Jia et al., 2019). Soil microbes are also important in maintaining the structure of soil, the decomposition of organic matter, the cycle of geochemistry, and the supply of nutrients (Roesch et al., 2007; Falkowski et al., 2008; Douterelo et al., 2010). As an important component of soil ecosystems, bacteria are essential for the carbon cycle and plant productivity (Johnson et al., 2003). The soil bacterial community composition and diversity are correlated to soil organic carbon transformation processes (Xiao et al., 2015). Fungi are important participants in the decomposition of organic matter and mineral components (Hollister et al., 2010). The magnitude of soil microbial availability is closely associated with the ecological environment they inhabit (Yan et al., 2018). Therefore, any change in the microbial communities of soil can lead to changes in various biochemical processes, thus affecting the stability of degraded ecosystems in mining areas, while also playing a vital role in the efficiency of ecological restoration. Over the course of a year, soil microbes face considerable variation in seasonal environmental factors, including temperature and humidity levels (López-Mondéjar et al., 2015) and the availability of nutrients (Du et al., 2018; Wang et al., 2018). Hence, soil microorganisms are often contingent on dynamic seasonal change characteristics. The seasonal variation in the biomass and structure of soil microorganisms also significantly differs among different ecosystems because the dominance of different environmental factors and the complexity of the comprehensive effects associated with various environmental factors vary among environments (Voříšková et al., 2014; López-Mondéjar et al., 2015).

In the damaged ecosystem of nonferrous mines, a large amount of heavy metals are disposed directly into soil along with waste rock, tailings, and other mineral dust in mining districts and their surrounding areas, which subsequently become the main source of environmental pollution. For instance, the Northern Copper Mine, the largest underground copper mine in China, has an annual output of greater than 7 million tons of ore (Jia et al., 2019). The extensive accumulation of tailings has led to severe pollution and the degeneration of the local ecological environment (Wang et al., 2010). Accordingly, a resolution to this problem is essential, and the best way to resolve this is to effectuate the reasonable and efficient ecological restoration of the copper tailings dam. The ecological functional recovery of soil is the key to such restoration as well as the sustainable development of terrestrial ecosystems (Wang et al., 2013; Jia et al., 2017). Thus, ecological restoration must also focus on soil fertility and the characteristics of dynamic change associated with microbial communities over the course of a whole year.

Although many studies have shown that anthropogenic activities can cause changes in soil microbial structure and diversity, there have been very few studies to date that have reported on the seasonal microbial community characteristics and its driving factors in copper tailings dams. Studies on the seasonal dynamics of the microbial community can improve our knowledge of microbial community ecology in contaminated environments and help to design and implement potential bioremediation strategies by which to address the progressively increasing impacts of xenobiotics in ecosystems (Hoostal and Bouzat, 2016). Therefore, the relationships between environmental conditions and seasonal variations of microbial communities within degraded tailings dam ecosystems warrant further investigation. This study will help to clarify the key environmental factors that affect soil microbial communities during ecological restoration processes in copper tailings dams. Moreover, our experiment explored effective biological indicators in each season during ecological restoration processes in a mining area, while revealing the regular energy transformation and material circulation patterns in soil.

To achieve this objective, we addressed the following questions: (1) How do soil bacterial and fungal communities vary with season changes? (2) What are the special functional bacterial and fungal in a copper tailings dam? (3) What are the dominant environmental factors that affect soil microbial structure and diversity over the course of a year? The aim of this study was to provide an ecological basis for the mechanisms of soil ecosystem restoration and degradation in different seasons, and to strengthen our understanding of soil property and microbial community biodiversity restoration in an environment subjected to pollution.

## Materials and Methods

### Site Description

**Figure 1:**
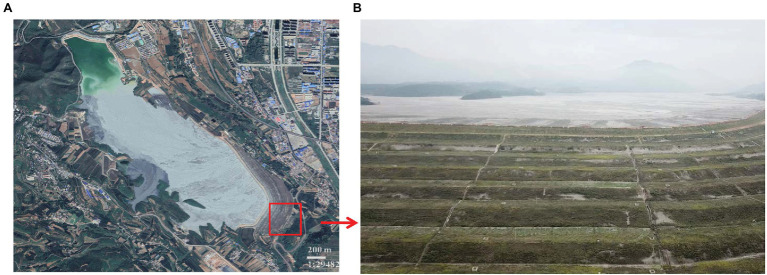
The panorama of study area **(A)** and the profile **(B)** of sub-dams in copper tailings dam.

Construction on the 18 river tailings (latitude 35°15′ ~ 35°17′ N, longitude 118°38′ ~ 111°39′ E) commenced in 1969, which is a constituent of the Northern Copper Mine, situated in the southern region of the province of Shanxi in China (Figure 1; Jia et al., 2019). Currently, this copper tailings dam comprises of 16 sub-dams. The study area is marked by four distinct seasons that are contingent on a continental “monsoon” climate. The duration of annual rainfall and temperature information of the study area was shown in Supplementary Table S1.

### Soil Sampling

The number No. 536 sub-dam of the 18 river tailings dam was selected in July 2017 for investigation. This sub-dam, in its 20th year of restoration, was used for sampling during all four seasons, namely, spring (March), summer (June), autumn (September), and winter (December; Jia et al., 2019). We randomly collected three soil samples from 1 × 1 m plots during each of the four seasons, and each sample plot was spaced greater than 50 m apart. Fresh soil samples were divided into two subsamples after being shifted using a 2 mm sieve. We stored the first subsample (4°C) prior to determining physiological and chemical properties, while we stored the second subsample (−20°C) prior to DNA extraction.

### Chemical Properties of Soil

Total carbon (TC), total nitrogen (TN), and total sulfur (TS) content of soil samples were measured using an elemental analyzer (vario EL/MACRO cube, Elementar, Hanau, Germany). Soil water (1:2.5 mass/volume) suspensions were shaken for 30 min prior to measuring soil pH. Gravimetric analysis was used to measure soil moisture. Ammonium nitrogen (NH_4_^+^-N), nitrate nitrogen (NO_3_^−^-N), and nitrite nitrogen (NO_2_^−^-N) in soil were measured using the Automatic Discrete Analyzer (CleverChem 380, DeChem-Tech, GmbH, Hamburg, Germany; Jia et al., 2018).

### Techniques Used for DNA Extraction, PCR Amplification, and MiSeq Sequencing

We used the E.Z.N.A.® Soil DNA Kit (Omega Bio-tek, Norcross, GA, USA) to extract soil microbial DNA using the manufacturer’s protocol. Extracted DNA was quantified using a NanoDrop ND-1000 UV-Vis Spectrophotometer (NanoDrop Technologies, Wilmington, DE, USA). Primers 515F (5'-GTGCCAGCMGCCGCGG-3') and 907R (5'-CCGTCAATTCMTTTRAGTTT-3') were used to amplify the V4-V5 hyper variable region of the 16S ribosomal RNA (rRNA) bacterial gene. Primers ITS1F (5'-CTTGGTCATTTAGAGGAAGTAA-3') and ITS2 (5'-GCTGCGTTCTTCATCGATGC-3') were used to determine the fungal internal transcribed spacer (ITS) gene copy number of all samples. Finally, sequencing was carried out at Shanghai Majorbio Bio-pharm Technology (Shanghai, China) using the MiSeq platform (Illumina, Inc., USA). The bacterial and fungal sequences have been deposited in the SRA of the NCBI database under the SRA accession: PRJNA600330 and PRJNA605500.

### Processing of Sequencing Data

Raw FASTQ files were demultiplexed and quality-filtered using QIIME (version 1.17) under the following criteria: 300-bp reads were truncated at any site receiving an average quality score of <20 over a 50-bp sliding window, and truncated reads shorter than 50-bp were discarded; exact barcode matching, two-nucleotide mismatch in primer matching, and reads containing ambiguous characters were removed; and only sequences that overlapped for more than 10-bp were merged according to their overlap sequence. Reads that could not be merged were discarded. Operational taxonomic units (OTUs) were clustered with a 97% similarity cutoff using UPARSE (version 7.1, http://drive5.com/uparse/), and chimeric sequences were identified and removed using UCHIME. The taxonomy of each 16S rRNA gene and ITS gene sequences respectively were analyzed using the Ribosomal Database Project (RDP) Classifier^1^ against the Silva (SSU128) 16S rRNA and unite 7.0 database with a confidence threshold of 70%.

### Statistical Analysis

SPSS Statistics version 20 was used to calculate the data derived from the analyses discussed above. Heatmapping of the top 10 genera in each sample was conducted using the R packages. We used non-metric multidimensional scaling (NMDS) and analysis of similarities (ANOSIM) analysis to investigate differences in bacterial and fungal community structure. Furthermore, we used redundancy analysis (RDA) or canonical correspondence analysis (CCA) to analyze relationships between microbial and environmental factors using Canoco 5.0 (Microcomputer Power, USA). Additionally, one-way ANOVA was used to analyze differences in environmental parameters as well as the alpha diversity (α-diversity) indices and the relative species abundance of the predominant bacterial and fungal species among the four seasons. Structural equation models (SEM) were analyzed using advanced mortar system (AMOS) version 13.0.

## Results

### Overall Taxonomic Distribution and Microbial Diversity

We extracted DNA from soil samples in the copper tailings dam during all four seasons. Moreover, we used the MiSeq platform to sequence 16S/ITS rRNA genes. For all samples, we obtained a total of 504,999 and 608,880 quality-filtered and chimera-checked 16S/ITS rRNA gene sequences with respective average lengths of 396 and 266 bp. Per sample, we recovered from 32,628 to 56,152 16S rRNA sequences and from 30,507 to 71,422 fungal ITS rRNA sequences. We also obtained a total of 2,982 bacterial OTUs and 810 fungal OTUs from soil samples based on 97% sequence similarity, which suggests that the sequencing data reflected most microbial diversity in the field. Across all samples, taxonomically classified bacterial OTUs were representative of 32 phyla, 79 classes, 171 orders, 317 families, and 5,629 genera, while fungal OTUs were representative of 6 phyla, 25 classes, 65 orders, 138 families, and 250 genera. The Venn diagram (Supplementary Figure S1) reveals that 1884 OTUs were common to bacterial communities, while 150 were common to fungal communities. We compared bacterial and fungal community richness estimators [i.e., Chao1 and abundance-based coverage estimator (ACE)] and diversity index (i.e., Shannon and Simpson) values among the different seasons (Table 1). Richness estimators suggested that bacterial community abundance in summer was significantly higher than corresponding values in winter. Shannon indexes showed that fungal communities in summer were higher than in autumn. In addition, Simpson indexes showed that fungal community diversity in summer was significantly higher than those of the other seasons, whereas bacterial community diversity was not significantly different among the four seasons (Table 1).

**Table 1:** Comparison between phylotype coverage and diversity estimators of soil microbial communities of the four seasons.

	Sample	Reads*^a^*	OTUs*^b^*	Coverage	Richness estimator		Diversity index	
					ACE	Chao1	Shannon	Simpson
Bacterial	Spring	31109A	1946A	0.987A	2240.01AB	2270.93AB	6.1608A	0.0141A
	Summer	23760B	2049A	0.978B	2443.79A	2447.91A	6.4742A	0.0035A
	Autumn	26604AB	1877A	0.982AB	2261.42AB	2249.20AB	6.2714A	0.0048A
	Winter	26438AB	1856A	0.984A	2145.00B	2152.60B	6.2772A	0.0052A
Fungal	Spring	68327a	268ab	0.9995a	289.50b	288.52b	2.984ab	0.097b
	Summer	36257b	327a	0.9980c	390.55a	383.70a	3.339a	0.087b
	Autumn	34340b	217b	0.9984b	268.37b	264.53b	2.421b	0.197a
	Winter	62343a	244ab	0.9994a	272.45b	273.60b	3.056ab	0.091b

a Provides reads after trimming and chimera removal. The coverage percentage, richness estimators (ACE and Chao1), and diversity indices (Shannon and Simpson) were calculated using the Good’s method and the Mothur program, respectively.

b Operational taxonomic units (OTUs) were defined at a 97% level of similarity. Significant differences between seasons are denoted with letters. (bacteria: A > B > C; fungal: a > b > c).

### Comparison Between Bacterial and Fungal Communities Among Seasons

**Figure 2:**
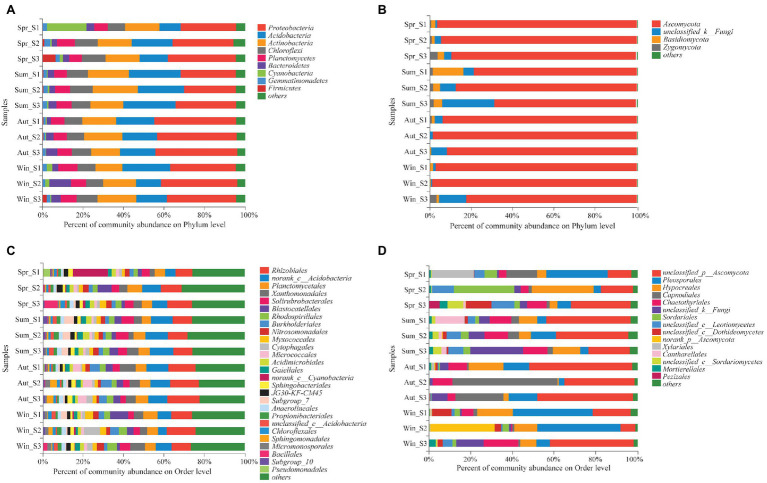
Relative abundance of the dominant phyla **(A,B)** and orders **(C,D)** of bacterial **(A,C)** and fungal **(B,D)** communities in soil (with average relative abundance >2%) among different seasons.

**Figure 3:**
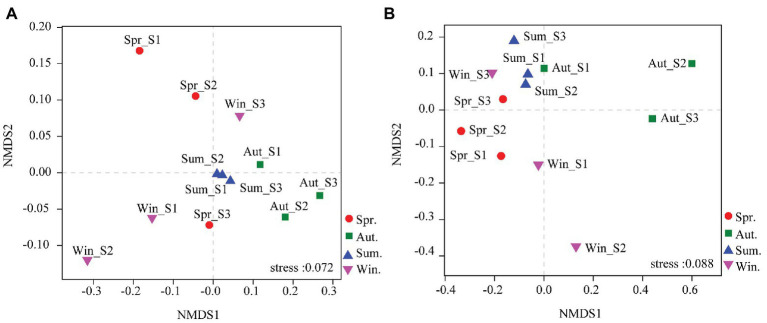
Non-metric multidimensional scaling (NMDS) ordination based on Bray-Curtis similarities of bacterial **(A)** and fungal **(B)** community compositions at four seasons. The results indicate a significant influence of season on bacterial and fungal community structures [assessed by a multivariate analysis of similarities (ANOSIM)].

**Figure 4:**
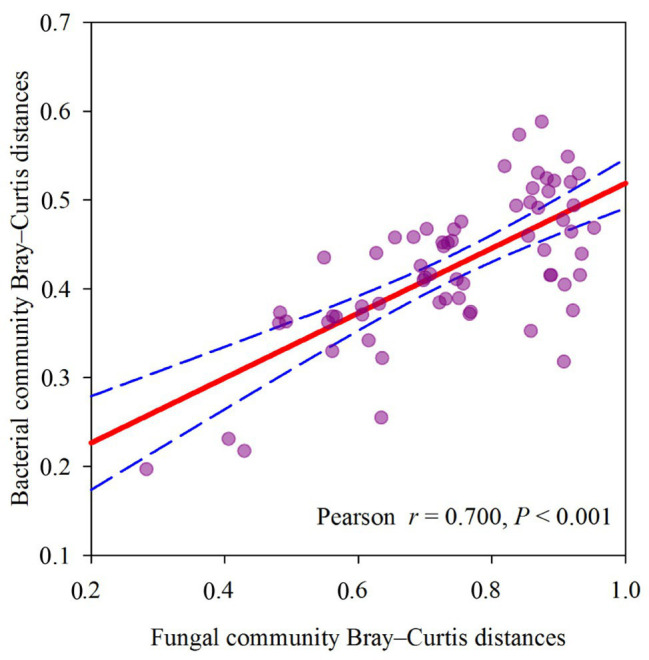
Relationship between soil fungal community Bray-Curtis distances and soil bacterial community Bray-Curtis distances, measured at plot scale across different seasons.

**Figure 5:**
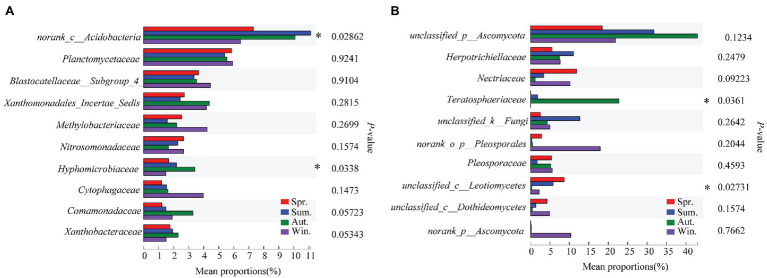
Relative abundances of top 10 bacterial **(A)** and fungal **(B)** families that showed significant differences among seasons. Kruskal-Wallis H test was used to evaluate the significance of differences between the indicated groups. ^*^ *p* < 0.05.

The dominant species comprising the bacterial and fungal communities were generally consistent among seasons; however, differences were found in the relative abundance. *Proteobacteria*, *Acidobacteria*, *Actinobacteria*, and *Chloroflexi* were the bacterial phyla with the highest relative abundances (Figure 2A). *Ascomycota* and *Basidiomycota* were the dominant fungal phyla (Figure 2B). At the order level, the dominant bacterial and fungal community members were *Rhizobiales* and *Pleosporales*, respectively (Figures 2C,D). Bray-Curtis dissimilarity based on NMDS and ANOSIM was determined to reveal the dissimilarity of microbial communities among each season (Figure 3). ANOSIM revealed significant differences in both bacterial (stress = 0.072; *R* = 0.435; *p* = 0.004) and fungal (stress = 0.088; *R* = 0.398; *p* = 0.006) community structure among the different seasons (Figure 3). Moreover, linear regression analysis revealed that fungal and bacterial community composition was significantly positively correlated (*p* < 0.001; Figure 4). The top 10 dominant microbial families are shown in Figure 5. For the bacterial community, we observed significant differences in norank_c_ *Acidobacteria* and *Hyphomicrobiaceae* at a family level (Figure 5A). For the fungal community, we observed significant differences in Teratosphaeriaceae and unclassified_c_*Leotiomycetes* at the family level during all four seasons (Figure 5B).

**Figure 6:**
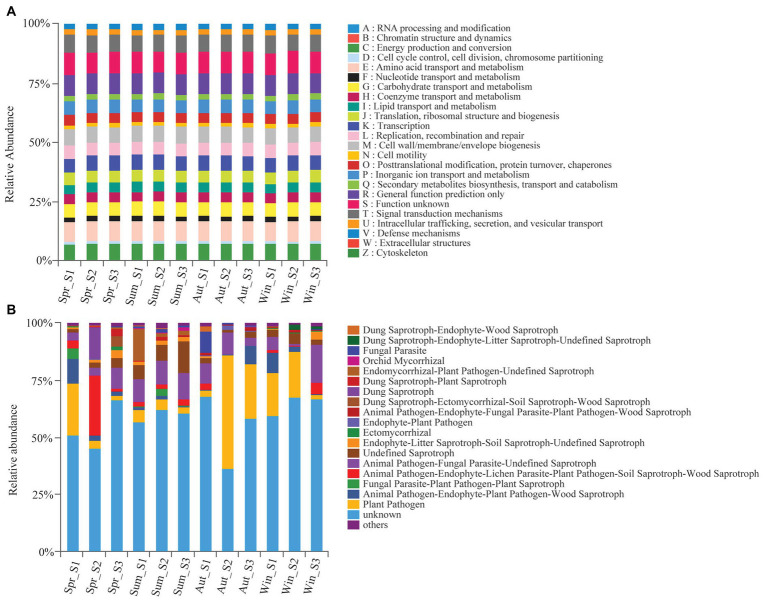
Variations in composition of bacterial **(A)** and fungal **(B)** functional groups inferred by the PICRUSt^10^ and FunGuild, respectively.

We used the PICRUSt and FUNGuild tools to better understand the important roles that bacteria and fungi play in copper tailings dams, respectively (Figure 6). Bacteria exhibited similar functional features during different seasons (Figure 6A). These functional features mainly included those related to energy production and conversion processes, amino acid transportation and metabolic processes, carbohydrate transportation and metabolic processes, and transcription processes as well as biogenetic and signal transduction processes associated with cell wall, membrane, and envelope mechanisms (Figure 6A). However, we observed significant differences in fungal functions among the four seasons. Plant pathogens accounted for 49.8% of the soil fungal OTUs detected in autumn (Figure 6B). Animal pathogen, endophyte-lichen parasite, plant pathogen, soil saprotroph, wood saprotroph (26.4%), and dung saprotroph (14.0%) were significantly higher in spring compared with the other three seasons, and parasitic fungi (9.4%) had the highest relative abundance in autumn (Figure 6B). Orchid mycorrhizae were only observed in summer. Ectomycorrhizae mainly occurred in winter and spring (Figure 6B).

### Microbial Community Structure and Environmental Variable Correlations

**Figure 7:**
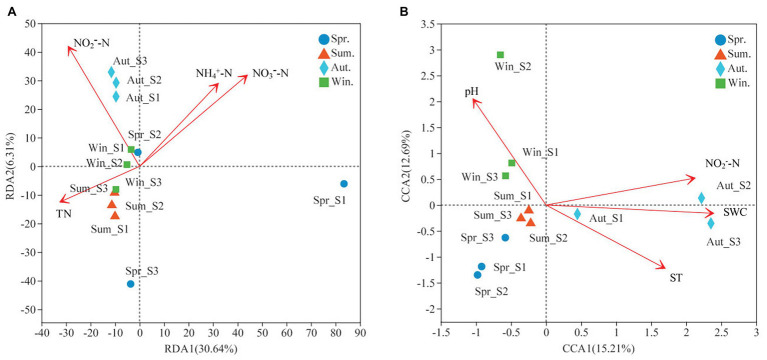
RDA or CCA of the soil bacterial **(A)** and fungal **(B)** community structure and environmental variables at four seasons. Only the environmental variables which were significantly correlated with RDA1, or CCA1 were shown in figures.

Soil characteristics varied seasonally (Table 2). Soil nutrients (TN and TC) were significantly higher in summer compared with the other seasons (*p* < 0.05), and pH was highest in winter (Table 2). Moreover, NO_2_^−^-N was significantly higher in autumn than in spring and summer (*p* < 0.05), but no significant differences were observed in winter. In autumn, the soil water content (SWC) was significantly lower than the other seasons (*p* < 0.05; Table 2). Our experiment evaluated the effects of these ecological factors on microbial community structure during different seasons (Figure 7). It was found that 36.95% of bacterial variation could be explained by soil properties (Figure 7A). Axis 1 of the RDA plot explained roughly 30.64% of variation, while Axis 2 explained a further 6.31%. The RDA results showed that NH_4_^+^-N, NO_3_^−^-N, NO_2_^−^-N, and TN were the major controls on the bacterial community structure (Figure 7A). Soil properties could explain 27.9% of the variability in fungal community structure (Figure 7B), where Axis 1 of the CCA plot explained 15.21% of the variability and Axis 2 explained a further 12.69%. Four soil characteristics were chosen for CCA after redundant variables were removed. As shown in Figure 7, SWC, soil temperature (ST), NO_2_^−^-N and pH significantly affected fungal community structure (Figure 7B).

**Table 2:** Soil chemical properties of copper tailings dam.

Physical and chemical factors	Spring	Summer	Autumn	Winter
SWC	10.52 ± 0.337b	0.84 ± 0.036c	86.16 ± 0.213a	9.10 ± 1.675b
pH	8.20 ± 0.028b	8.20 ± 0.017b	8.11 ± 0.029b	8.89 ± 0.038a
NH_4_^+^-N/mg·kg^−1^	0.40 ± 0.109	0.23 ± 0.086	0.43 ± 0.075	0.19 ± 0.105
NO_3_^−^-N/mg·kg^−1^	0.15 ± 0.064	0.07 ± 0.008	0.13 ± 0.007	0.11 ± 0.018
NO_2_^−^-N/mg·kg^−1^	0.01 ± 0.000b	0.01 ± 0.000b	0.01 ± 0.002a	0.01 ± 0.001ab
TN/g·kg^−1^	0.04 ± 0.003b	0.08 ± 0.005a	0.05 ± 0.005b	0.05 ± 0.006b
TC/g·kg^−1^	0.92 ± 0.033b	6.10 ± 0.969a	1.03 ± 0.041b	1.04 ± 0.116b
C/N	24.48 ± 2.024b	81.02 ± 16.746a	22.06 ± 1.474b	21.37 ± 2.060b
TS/g·kg^−1^	0.06 ± 0.006	0.46 ± 0.295	0.14 ± 0.017	0.05 ± 0.002
ST/°C	16.83 ± 1.354b	24.27 ± 0.318a	26.87 ± 0.484a	8.10 ± 1.002c
Salinity/mg·L^−1^	14.33 ± 3.712b	0.00 ± 0.000c	31.67 ± 1.202a	4.00 ± 1.000c
EC/μs·cm^−1^	26.33 ± 6.766b	0.00 ± 0.000c	57.67 ± 1.667a	7.33 ± 1.856c

Values represent mean with standard error in parenthesis. Significant differences between sites (Duncan test, *p* < 0.05) are denoted with letters (a > b > c). SWC, soil water content; NH_4_^+^-N, ammonium nitrogen; NO_3_^−^-N, nitrate nitrogen; NO_2_^−^-N, nitrite nitrogen; TN, total nitrogen; TC, total carbon; TS, total sulfur; C/N, the ratio of carbon and nitrogen; ST, soil temperature; EC, electrical conductivity.

**Figure 8:**
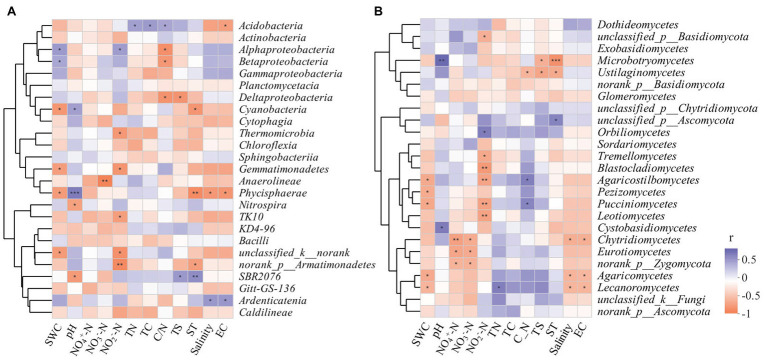
Spearman correlation heatmap of the top 25 soil bacterial **(A)**, fungal **(B)** classes and soil properties. X and Y axis are environmental factors and classes. R in different colors to show, the right side of the legend is the color range of different r values. ^*^ *p* < 0.05; ^**^ *p* < 0.01; ^***^ *p* < 0.001.

**Figure 9:**
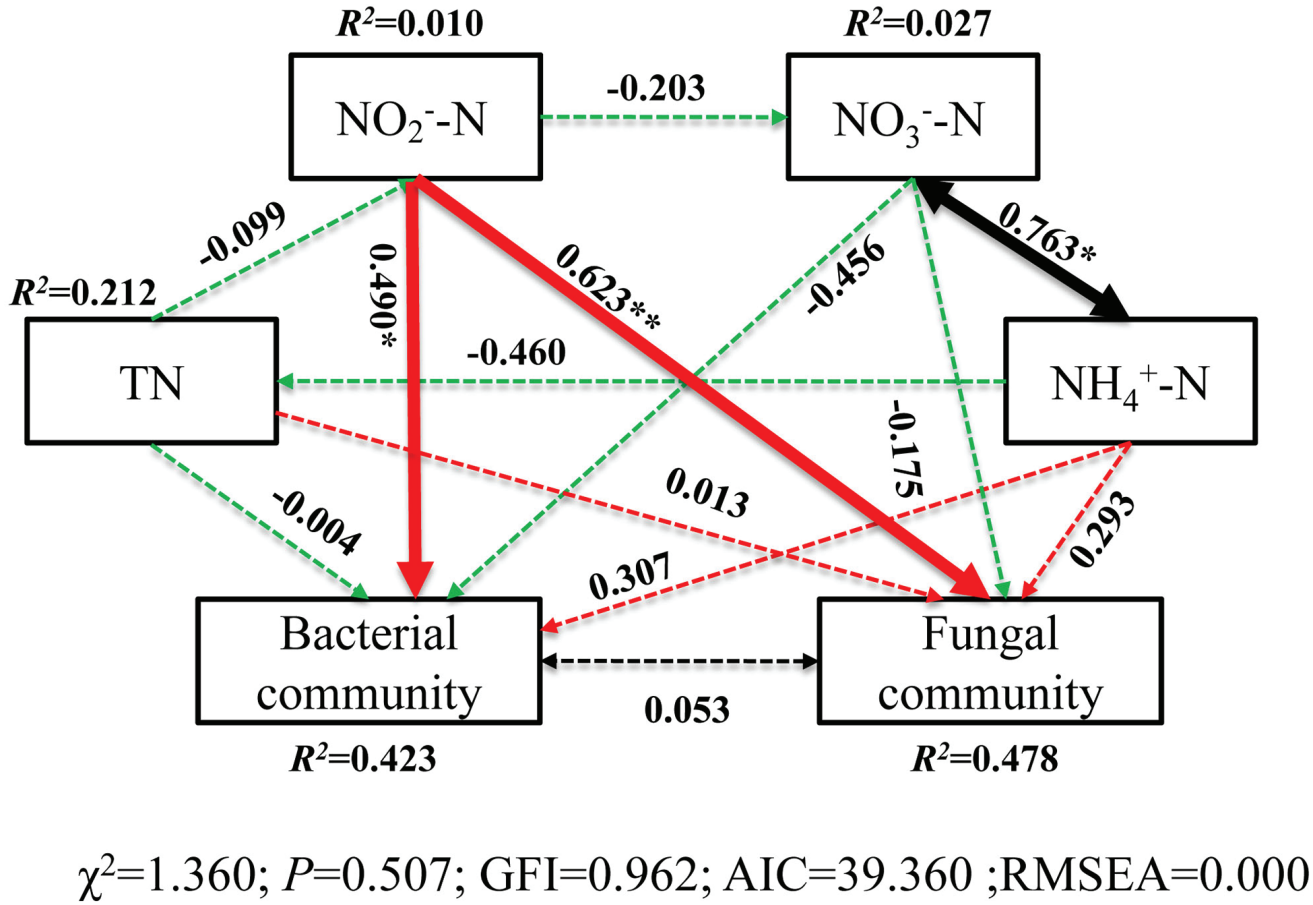
Structural equation model (SEM) illustrating the effects of soil properties on microbial communities. Continuous and dashed arrows represent the significant and non-significant relationships, respectively. Adjacent numbers that are labeled in the same direction as the arrow represents path coefficients, and the width of the arrow is in proportion to the degree of path coefficients. Green and red arrows indicate positive and negative relationships, respectively. Black arrows indicate integrations. *R* ^2^ values indicate the proportion of variance explained by each variable. Significance levels are denoted with ^*^ *p* < 0.05 and ^**^ *p* < 0.01. Standardized total effects (direct plus indirect effects) calculated by the SEM are displayed below the SEM. The low chi-square (*χ* ^2^), non-significant probability level (*p* > 0.05), high goodness-of-fit index (GFI > 0.90), low Akaike information criteria (AIC), and low root-mean-square errors of approximation (RMSEA < 0.05) listed below the SEMs indicate that our data matches the hypothetical models.

The correlation heatmap (Figure 8) showed that the relationship between microbial composition and environmental factors differed between bacterial and fungal communities. For the bacterial community, strains *Nitrospira* and *SBR2076* exhibited significant negative correlations to pH, whereas Phycisphaerae and *Cyanobacteria* were positively correlated to pH. Phycisphaerae exhibited highly significant negative correlations to SWC, ST, salinity and EC (Figure 8A). *Acidobacteria* were both significantly and positively correlated to soil TN, TC, and C/N (Figure 8A). For the fungal community, both *Cystobasidiomycetes* and *Microbotryomycetes* abundance was positively correlated to pH. Both *Ustilaginomycetes* and *Microbotryomycetes* were negatively correlated to soil sulfur and ST (Figure 8B). We constructed a SEM to further quantify the contribution of driving factors to microbial communities (Figure 9). Soil NO_2_^−^-N was the dominant factor influencing both the bacterial and fungal communities. Moreover, interactions between NO_3_^−^-N and NH_4_^+^-N were direct and significant; however, no bacterial and community interactions were observed in soil (Figure 9).

## Discussion

### Soil Microbial Diversity in a Copper Tailings Dam

Ecological soil functions are based on soil microbial communities. Soil microbes affect soil nutrient cycling and regulation, and they can be used as indicators of soil functions through their participation in soil organic matter’s associated decomposition and mineralization processes (Romaniuk et al., 2011). In this study, bacterial richness, fungal richness, and the Shannon index reached their maximum in summer, indicating that this specific season exhibited the highest overall soil microbial activity. This could be because plant photosynthesis was strong in summer. In other words, photosynthetic products enter the soil through root systems, and these products are used by microorganisms as a nutrient source, which promote microbial growth and reproduction (López-Mondéjar et al., 2015). Another possible explanation is that seasonal changes in ST also affect microbial growth. The copper tailings area investigated for this study is warm in summer and cold in winter. In general, microbial activity and species richness increase with an increase in temperature (Sierra et al., 2015), whereas microbial activity decreases with a decrease in temperature (Lipson et al., 2002). This is probably because of a reduction in the lipid fluidity of microbial membranes, resulting in the freezing of intracellular fluids as well as the rupture and death of cells (Yergeau and Kowalchuk, 2008). Therefore, high temperatures in summer provide a relatively stable environment for soil microbial growth.

### Soil Microbial Community Composition and Function

The dominant bacterial phyla and class in the copper tailings dam were *Proteobacteria*, *Acidobacteria*, *Actinobacteria*, and *Chloroflexi*, which are consistent with the findings of Lauber et al. (2009). Our study found that seasonal variation significantly affected the dominant soil bacteria in the copper tailings dam. The relative abundance of *Bacteroidetes* was lower than *Acidobacteria*, and the relative abundance of *Bacteroidetes* was highest in winter (Figure 2). Margesin and Schinner (1994) reported that *Bacteroidetes* are cold-tolerant bacteria that have unique physiological mechanisms to resist low temperatures, which enables members of this phylum to subsist in cold environments. *Acidobacteria* are an oligotrophic bacterial phylum, while *Bacteroidetes* have entropic characteristics (Mccaig et al., 1999; Fazi et al., 2005; Dion, 2008). The results from this study could also have been caused by soil nutrient depletion in the copper tailings dam, and soil nitrogen becoming a limiting factor in the study area (Table 2).

*Ascomycota* and *Basidiomycota* were the dominant fungal phyla during all four seasons (Figure 2B). They were the main decomposers in soil, while also playing a vital role in nutrient cycling. The fungal community exhibited clear seasonal change characteristics, which is consistent with the findings of Andreetta et al. (2012), which showed that microbial communities exhibited seasonal variability resulting from seasonal variation in environmental conditions. The relative abundance of *Basidiomycota* in the copper tailings dam was highest in summer, which is consistent with the findings of Chen et al. (2016). This is because the energy that flows from roots to soil promotes plant growth and subsequently variations in mycorrhizal fungi that surround the root zone area in summer. Most *Basidiomycota* can form mycorrhizae with roots, while only a limited number of *Ascomycota* members can form mycorrhizae. Moreover, most *Ascomycota* are saprophytes. In this study, the relative abundance of *Ascomycota* was high during all four seasons. The response of *Ascomycota* to environmental stress was relatively stable, because it was the dominant phylum in multi-contaminated and non-contaminated ecosystems, as shown by Ventorino et al. (2016).

The fungal community exhibited significant differences in functional features among the four seasons, but no clear differences in bacterial functional groups were observed in this study. The different seasonal responses between the community functions of soil bacteria and soil fungi could result from the lower sensitivity that fungi have to all environmental changes. This is because fungal generation is generally slower than that of bacteria, and thus responds more slowly to soil disturbances (Fiorentino et al., 2018). József et al. (2015) reported that the abundance of saprophytic fungi increases with an increase in temperature, while the abundance of ectomycorrhizal fungi generally decreases with an increase in temperature. Our study also showed that the abundance of saprophytic fungi was high in summer, and the abundance of symbiotic fungi (i.e., ectomycorrhizal fungi) was high in winter (Figure 6). This could be because rising temperatures result in an increase in microbial abundance along with organic matter decomposition in summer (Sistla et al., 2013). The role that saprophytic fungi play in organic matter decomposition is particularly important. Competition exists between functional fungal groups. Pathogenic and saprophytic soil fungi were closely bound to roots throughout both the non-growing and growing seasons. Ectomycorrhizal fungi (such as *Thelephoraceae* and *Tuberaceae*) gradually replaced other fungal groups and ultimately became the dominant fungal communities during the growing season (Jumpponen et al., 2010). However, the relative abundance of symbiotic fungi increased in winter in this study. This is likely to be because soil samples, excluding root tips or plant samples, were collected specifically for fungal community analysis. Moreover, spores and mycelia in soil were less affected by plant development.

### Soil Microbial Community and Soil Factor Relationships

In this study, RDA analysis showed that the available soil nitrogen content significantly affected bacterial community structure (i.e., NH_4_^+^-N, NO_3_^−^-N, and NO_2_^−^-N). Soil available nitrogen was a leading factor in the seasonal variation of soil bacterial communities. This suggested that the adaptability of bacterial communities to soil nutrient availability varied, resulting in seasonal bacterial community variation. Hu et al. (2002) reported that the relative abundance of soil microbes was significantly positively correlated to soil nutrients. Increases in soil nutrients will promote the growth of microorganisms and thus, to a certain extent, microbial abundance represents the quality of soil biological fertility. Soil pH affects soil microbial biological activities by influencing physiochemical soil properties and the composition of the soil matrix. In our study, we found that Phycisphaerae and *Cyanobacteria* were positively correlated to pH. *Cyanobacteria* are known to colonize plant roots (Gantar et al., 2010; Lundberg et al., 2012), which can encourage plant growth (Prasanna et al., 2009). Meanwhile, *Cyanobacteria*, owing to its N-fixation ability, are a key source of inorganic N for plants (Franche et al., 2009). *Alphaproteobacteria* and *Betaproteobacteria* were positively correlated to soil C/N, and were significant negative correlations to SWC (Figure 8A). It has reported that *Proteobacteria* was the most stress-tolerant phylum under conditions of heavy soil contamination (Eva-Maria et al., 2011). Moreover, *Proteobacteria* have previously exhibited considerable diversity in morphology, physiology, and metabolic processes (Zavarzin et al., 1991), suggesting that this bacterial can adapt to different environments by physiological and metabolic regulation processes. We also found *Acidobacteria* were positively correlated to soil TC, TN, and C/N (Figure 8A). *Acidobacteria* can degrade complex lignin and cellulose to provide soil nutrients (Lynd et al., 2002; Pankratov et al., 2011).

Furthermore, studies have shown that variation in both long-term and short-term SWC can alter the soil fungal community structure (Pasternak et al., 2013; Evans et al., 2014; Hartmann et al., 2017; Engelhardt et al., 2018). Our study found that ST, SWC, NO_2_^−^-N, and soil pH were all important factors influencing soil fungal community variation (Figure 7). Moreover, *Agaricomycetes* and *Lecanoromycetes* were negatively correlated to SWC, salinity, and EC (Figure 8B). *Agaricomycetes* act as important decomposers, producing both hydrogen peroxide and enzymes, resulting in the degradation of complex plant compounds, such as cellulose and lignin (Kameshwar and Qin, 2016). Soil fungi possess strong decomposition abilities. The effects of soil fungi on labile organic carbon mainly depend on a variety of enzymes that decompose organic matter, particularly recalcitrant organic matter. We further found that *Basidiomycota* and *Ascomycota* were the dominant fungal phyla during all four seasons. These two fungal phyla possess critical genes that can encode cellulose decomposition enzymes and promote carbon conversion processes (Hannula et al., 2012; Bastida et al., 2013). *Basidiomycota* mainly depend on plant litter or soil organic matter as their primary carbon source, and members of this phylum participate in soil carbon transformation processes (Jiang et al., 2006).

In future studies, *Cyanobacteria*, *Proteobacteria*, *Acidobacteria*, *Basidiomycota*, and *Ascomycota* could be inoculated into plant or soil in the process of bioremediation, and studied their effects on plant growth and physiology, so as to better restore contaminated sites *via* a combined microbial and plant interaction. This combined investigation of seasonal microbial communities in a copper tailings dam offers an opportunity to further elucidate on microbial adaptations under ecological restoration in mining areas, allowing us to better understand the ability of the microbial community to colonize soil ecosystems by means of natural biofertility. More studies are still required to further understand the resistance and tolerance of such species as well as the molecular mechanisms involved in the adaptation of spontaneous microbial biodegraders in the contaminated soil of copper tailings dams.

## Data Availability Statement

The datasets presented in this study can be found in online repositories. The names of the repository/repositories and accession number(s) can be found in the article/Supplementary Material.

## Author Contributions

TJ conceived and designed the experiments. TG, YY, and RW performed the experiments. BC contributed new reagents. TJ wrote the manuscript. All authors contributed to the article and approved the submitted version.

## Conflict of Interest

The authors declare that the research was conducted in the absence of any commercial or financial relationships that could be construed as a potential conflict of interest.

## Supplementary Material

The Supplementary Material for this article can be found online at: https://www.frontiersin.org/articles/10.3389/fmicb.2020.01574/full#supplementary-material.

## Footnotes

^1^http://rdp.cme.msu.edu/
