# Supplementary material for: Seasonal Microbial Community Characteristic and Its Driving Factors in a Copper Tailings Dam in the Chinese Loess Plateau
Source: Front Microbiol. 2020 Jul 10;11:1574. doi: 10.3389/fmicb.2020.01574 (PMC7366875; doi:10.3389/fmicb.2020.01574)
Supplement: Supplementary file 1 [file Data_Sheet_1.PDF]

Seasonal microbial community characteristic and its driving factors in a copper tailings dam in the Chinese Loess Plateau

Tong Jia*, Tingyan Guo, Yushan Yao, Ruihong Wang, Baofeng Chai

Shanxi Key Laboratory of Ecological Restoration on Loess Plateau, Institute of Loess Plateau, Shanxi University, Taiyuan 030006, P.R. China;

*Corresponding author. Email: jiatong@sxu.edu.cn

Address: No. 92 Wucheng Road, Xiaodian district, Taiyuan, Shanxi 030006, P.R. China. Tel.: +86-155-1369-4458

Table S1. The duration annual rainfalls and temperatures in Yuanqu county of Yuncheng city, Shanxi.

Month	Average precipitation (mm)	Maximum rainfall (mm)	Average temperature (℃)
Jan	8.1	36.5	-0.4
Feb	11.0	44.6	2.4
Mar	23.1	60.1	7.4
Apr	31.3	121.7	14.6
May	59.3	170.1	19.7
Jun	67.9	198.7	24.1
Jul	141.6	472.7	25.6
Aug	118.5	372.0	24.1
Sep	86.5	242.4	19.6
Oct	45.5	117.4	13.9
Nov	20.1	81.1	7.2
Dec	7.6	35.4	1.6

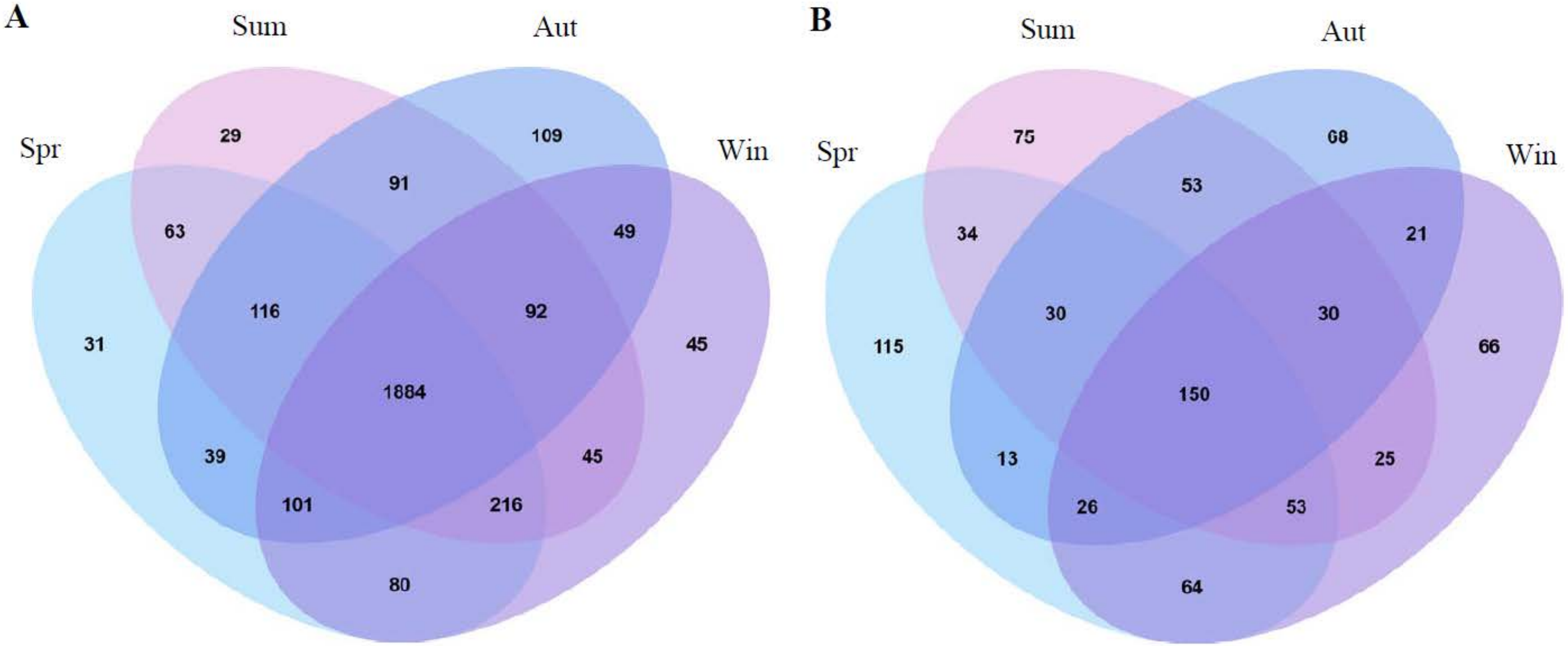

Fig. S1 Venn diagram for bacterial (A) and fungal (B) communities among different seasons. Numbers indicated shared unique operational taxonomic units (OTUs) at 0.03 dissimilarity distances after removing singletons involved.
